# 52 Use of corticosteroids in childhood systemic lupus erythematosus: experience from a pediatric rheumatology center

**DOI:** 10.1093/rheumatology/keac496.048

**Published:** 2022-10-07

**Authors:** Ourida Gacem, Kahina Abba, Nawal Mansouri, Nadia Ayad, Zoulikha Zeroual, Zakia Arrada, Moussa Achir, Mohamed Samir Ladj

**Affiliations:** Pediatrics Department Hospital Djillali Belkhenchir Birtraria Algiers Algeria

**Keywords:** *Systemic lupus erythematosus*, Corticosteroids, Children

## Abstract

**Background:**

Childhood-onset Systemic Lupus Erythematosus (cSLE) is a rare but severe multisystem autoimmune/inflammatory disease that can affect any organ system and cause significant damage, disability and/or death. Corticosteroids are one of the important therapeutic weapons for childhood Systemic Lupus Erythematosus (SLE). Prednisone doses are increased during flare-ups of lupus disease, and then progressively reduced to reach a threshold dose in order to avoid the harmful effects of this treatment in growing children. The oral route is by far the most used over prolonged periods, however corticosteroid boluses are recommended in the treatment of severe acute attacks of the disease.

**Objective:**

Assess the use of corticosteroids in childhood systemic lupus erythematosus (SLE) and the risk of steroid-related damage.

**Methods:**

Patients with childhood -onset SLE (*n* = 83) treated at paediatric rheumatology centre were followed for 36 months. Information on medication use, disease activity as measured by SLEDAI was collected.

**Results:**

Eighty-three (83) patients were studied. Female: male ratio was 1:49. Mean ages at lupus onset and diagnosis were respectively: 10, 12 (±3,88_ and 11, 3 (±3,62) years. Corticosteroids were used in 92% of cases in oral form and on 37% of cases as a bolus of high dose intravenous methylprednisolone. The indication of corticoids bolus was significantly related to severe forms (*p* = 0,001), high disease activity (*p* = 0, 00008), and macrophage activation Syndrome (*p* = 0, 0006). The doses varied between 0.5–1 g/1.73m2. The mean initial disease activity in these patients was 30 ± 10, while that found after taking the treatment was 12.9 ± 15. The decrease was statistically significant (*p* = 0, 000001). More than half of our patients were on high dose oral corticosteroids (2 mg/kg/d) and only 1/5 of the patients benefited from a dose of (1 mg/kg/day). The use of corticosteroids has been associated with significant side effects, including growth delay (22%), cushingoid obesity (58%), stretch marks (49%), osteoporosis (33%), femoral head osteonecrosis (1%), infections (54%). The association of certain side effects with a high dose of oral corticosteroids (2 mg/kg/day) was significant in this case cushingoid obesity (*p* = 0, 0003), stretch marks (*p* = 0.014).

**Conclusion:**

Corticosteroids are the mainstay of therapy for prompt control of acute disease related inflammation in patients with SLE; they remain the first-line treatment for significant lupus manifestations, especially severe forms and life-threatening situations Higher dosage and longer duration of therapy, which are common in SLE, result in greater risk of adverse effects. Their use wisely is the guarantee of a better response and the reduction of deleterious effect.

